# Niclosamide sensitizes triple-negative breast cancer cells to ionizing radiation in association with the inhibition of Wnt/β-catenin signaling

**DOI:** 10.18632/oncotarget.9704

**Published:** 2016-05-30

**Authors:** Lina Yin, Yun Gao, Xuxia Zhang, Jing Wang, Defang Ding, Yaping Zhang, Junxiang Zhang, Honghong Chen

**Affiliations:** ^1^ Department of Radiation Biology, Institute of Radiation Medicine, Fudan University, Shanghai, China

**Keywords:** niclosamide, radiosensitization, Wnt/β-catenin signaling, triple-negative breast cancer

## Abstract

Triple-negative breast cancer (TNBC) is one of the most difficult breast cancers to treat because there is no targeted treatment, and conventional cytotoxic chemotherapy followed by adjuvant radiation therapy is the standard of care for patients with TNBC. We herein reported that ionizing radiation (IR) induced Wnt3a, LRP6 and β-catenin expression and consequently activated Wnt/β-catenin signaling in TNBC MDA-MB-231, MDA-MB-468 and Hs578T cells. Moreover, depletion of β-catenin by shRNA sensitized TNBC cells to IR, whereas treatment of Wnt3a protein or overexpression of β-catenin resulted in radioresistance of TNBC cells. Niclosamide, a potent inhibitor of Wnt/β-catenin signaling, not only inhibited constitutive Wnt/β-catenin signaling, but also blocked IR-induced Wnt/β-catenin signaling in TNBC cells. In addition, niclosamide sensitized TNBC cells to IR, prevented Wnt3a-induced radioresistance, and overcame β-catenin-induced radioresistance in TNBC cells. Importantly, animals treated with the combination of niclosamide and γ-ray local tumor irradiation had significant inhibition of MDA-MB-231 tumor growth compared with treated with local tumor irradiation alone. These findings indicate that Wnt/β-catenin signaling pathway plays an important role in the development of radioresistance of TNBC cells, and that niclosamide had significant radiosensitizing effects by inhibiting Wnt/β-catenin signaling in TNBC cells. Our study also provides rationale for further preclinical and clinical evaluation of niclosamide in TNBC management.

## INTRODUCTION

Breast cancer is the most common cancer in women worldwide and is the second leading cause of death in women in many wealthy countries. Triple-negative breast cancer (TNBC), which lacks estrogen receptor (ER) and progesterone receptor (PR) expression as well as human epidermal growth factor receptor 2 (HER2) amplification, is one of the most difficult breast cancers to treat because there is no targeted treatment [1, 2]. Wnt/β-catenin signaling plays an important role in embryonic development and stem cell maintenance, and its aberration activation can lead tumor formation [3–5]. Activation of Wnt/β-catenin signaling, as defined by β-catenin nuclear expression and overexpression of the Wnt/β-catenin target cyclin D1, is associated with a poorer prognosis in breast cancer patients [6]. Recent studies further demonstrated that Wnt/β-catenin signaling activation was preferentially found in TNBC [7, 8], such that Wnt receptor frizzled-7 (FZD7) and Wnt co-receptor LRP6 were found to be up regulated in TNBC [9–11]. Therefore, LRP6 and Fzd7 could serve as novel therapeutic targets for the treatment of TNBC [12].

With no targeted treatments currently available, conventional cytotoxic chemotherapy followed by adjuvant radiation therapy is the standard of care for patients with TNBC. Studies have demonstrated that patients with TNBC who received standard adjuvant chemotherapy plus radiation therapy had decreased risk of locoregional recurrence and increased overall survival in comparison to those that received chemotherapy alone [13–15]. However, many breast cancer patients fail to respond to conventional radiotherapy/chemotherapy, leading to tumor recurrence [1, 2]. Ionizing radiation (IR) treatment can activate multiple signaling pathways in tumor cells, which are associated with radioresistance in many types of cancer [16]. Studies have demonstrated that IR can selectively enrich for breast cancer stem cells with activated Wnt/β-catenin signaling, which mediates radioresistance of breast cancer cells [17–19]. Therefore, Wnt inhibitors could have a therapeutic potential to overcome radioresistance of TNBC.

Niclosamide, an antihelminthic drug, has been approved for use in humans for over 50 years [20, 21]. Recent studies have identified niclosamide as a potential anticancer agent by targeting multiple intracellular signaling pathways [22]. It has been demonstrated that niclosamide inhibits Wnt/β-catenin signaling by enhancing Wnt receptor Fzd1 internalization, promoting Wnt co-receptor LRP6 degradation, and inhibiting β-catenin/T-cell factor (TCF) complex formation [23–26]. Notably, niclosamide inhibited Wnt/β-catenin signaling by enhancing LRP6 degradation in TNBC cells and suppressed TNBC cell proliferation *in vitro* and tumor growth *in vivo* [25, 27]. In the present study, we demonstrated that IR activated Wnt/β-catenin signaling in TNBC cells, and that activation of Wnt/β-catenin signaling resulted in radioresistance of TNBC cells. Moreover, niclosamide had significant radiosensitizing effects by suppressing Wnt/β-catenin signaling in TNBC cells, providing experimental evidence that combined treatment with niclosamide and radiation is a potential new treatment for TNBC patients.

## RESULTS

### IR induces activation of Wnt/β-catenin signaling in TNBC cells

**Figure 1:**
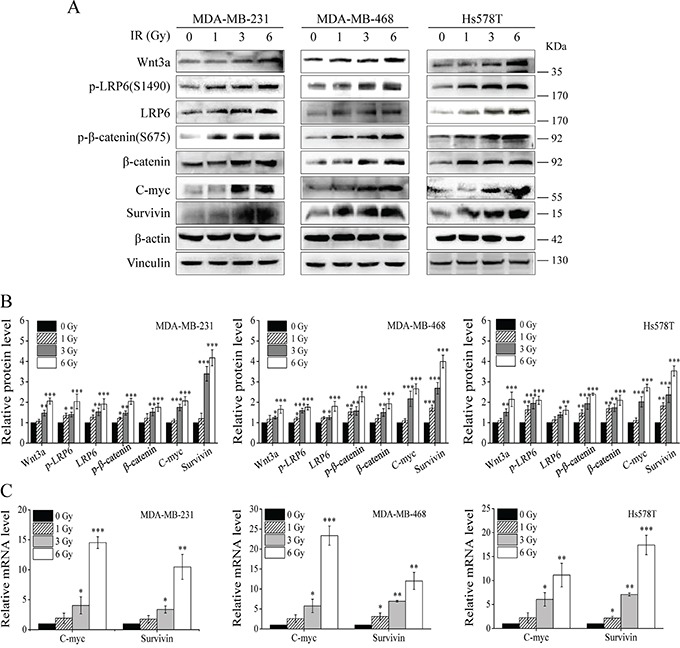
IR induces activation of Wnt/β-catenin signaling in TNBC cells **A.** MDA-MB-231, MDA-MB-468 and Hs578T cells were irradiated with indicated doses of γ-ray IR. After 6 h incubation, levels of Wnt3a, p-LRP6 (S1490), LRP6, p-β-catenin (S675), β-catenin, C-myc, survivin were analyzed by Western blotting. All the samples were also probed with anti-β-actin and anti-vinculin antibody to verify equal loading. **B.** The bands of each protein in above experiments were quantified by densitometric analysis, and normalized to the corresponding level of β-actin. **C.** MDA-MB-231, MDA-MB-468 and Hs578T cells were irradiated with indicated doses of γ-ray IR. After 6 h incubation, the mRNA levels of C-myc and survivin were examined by real-time RT-PCR. Values are averages of three independent experiments with the standard deviations indicated by error bar. ^*^P<0.05, ^**^P<0.01, ^***^P<0.001 versus corresponding non-irradiated cells.

It has been reported that IR enriches stem cell-like breast progenitor cells with highly activated Wnt/β-catenin signaling [17]. To test whether IR activates Wnt/β-catenin signaling in TNBC cells, we performed Western blotting to examine Wnt/β-catenin signaling in TNBC MDA-MB-231, MDA-MB-468 and Hs578T cells. As shown in Figure 1A and 1B, IR induced Wnt3a expression, Wnt co-receptor LRP6 expression and phosphorylation, and β-catenin expression in TNBC cells. It has been demonstrated that the activity of Wnt/β-catenin signaling can be enhanced by phosphorylation of β-catenin at Ser675 [28, 29]. We also found that IR induced β-catenin phosphorylation at Ser675 (Figure 1A and 1B). In addition, the transcript and protein levels of Wnt targets C-myc and survivin were significantly increased after IR in TNBC cells (Figure 1A, 1B and 1C). Together, these results indicate that IR activates Wnt/β-catenin signaling in TNBC cells.

It was recently reported that IR increased β-catenin protein expression, but did not change the β-catenin mRNA level in osteoblastic cells [30]. We performed real-time RT-PCR to test whether IR regulates the expression of Wnt3a, LRP6 and β-catenin at the transcriptional level in TNBC cells. As shown in Supplemental Figure S1, mRNA levels of Wnt3a, LRP6 and β-catenin were not significantly changed after IR in MDA-MB-231, MDA-MB-468 and Hs578T cells.

### Niclosamide inhibits IR-induced activation of Wnt/β-catenin signaling in TNBC cells

**Figure 2:**
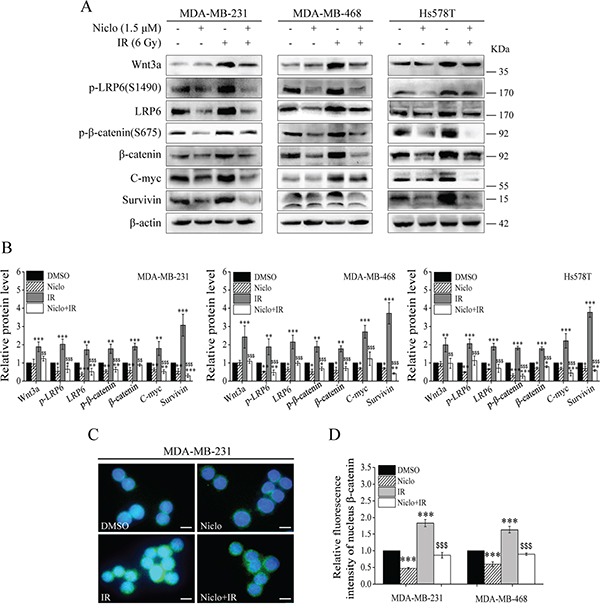
Niclosamide inhibits Wnt/β-catenin signaling in TNBC cells **A, B.** MDA-MB-231, MDA-MB-468 and Hs578T cells were treated with γ-ray IR (6 Gy) in the absence or presence of niclosamide (1.5 μM) for 24 h. Levels of Wnt3a, p-LRP6 (S1490), LRP6, p-β-catenin (S675), β-catenin, C-myc and survivin were analyzed by Western blotting. All the samples were also probed with anti-β-actin antibody to verify equal loading. The bands of each protein were quantified by densitometric analysis, and normalized to the corresponding level of β-actin. Values are averages of three independent experiments with the standard deviations indicated by error bar. **C, D.** MDA-MB-231 and MDA-MB-468 cells were treated with the same conditions as in (A). The cells were fixed, permeabilized and immunolabeled for β-catenin. β-catenin nuclear staining was detected by fluorescent microscopy (1000×magnification; Scale bar, 10 μm). The relative mean fluorescence intensity of nuclear β-catenin staining was calculated out of a total number of 200 cells per sample. Values are averages of three independent experiments with the standard deviations indicated by error bars. ^*^P<0.05, ^**^P<0.01, ^***^P<0.001 versus corresponding cells treated with DMSO control; ^$$^p<0.01, ^$$$^p<0.001 versus corresponding cells treated with IR alone. Niclo, niclosamide.

It has been demonstrated that niclosamide inhibits Wnt/β-catenin signaling by suppressing LRP6 expression in TNBC cells [25]. Therefore, we tested whether niclosamide is able to inhibit IR-induced Wnt/β-catenin signaling in TNBC cells. As expected, niclosamide at 1.5 μM in the absence or presence of 6 Gy IR suppressed the levels of LRP6 expression, LRP6 phosphorylation, β-catenin phosphorylation at Ser675, β-catenin expression, and expression of Wnt targets C-myc and survivin in MDA-MB-231, MDA-MB-468 and Hs578T cells (Figure 2A and 2B). It was noted that niclosamide markedly suppressed IR-induced Wnt3a expression in TNBC cells, although it had no obvious effects on endogenous Wnt3a expression (Figure 2A and 2B). Moreover, immunofluorescence staining demonstrated that niclosamide significantly decreased IR-induced β-catenin nuclear localization in MDA-MB-231 and MDA-MB-468 cells (Figure 2C and 2D). Together, these results indicate that niclosamide not only inhibited constitutive Wnt/β-catenin signaling, but also blocked IR-induced Wnt/β-catenin signaling in TNBC cells.

### Niclosamide enhances IR-induced apoptosis and sensitizes TNBC cells to IR

**Figure 3:**
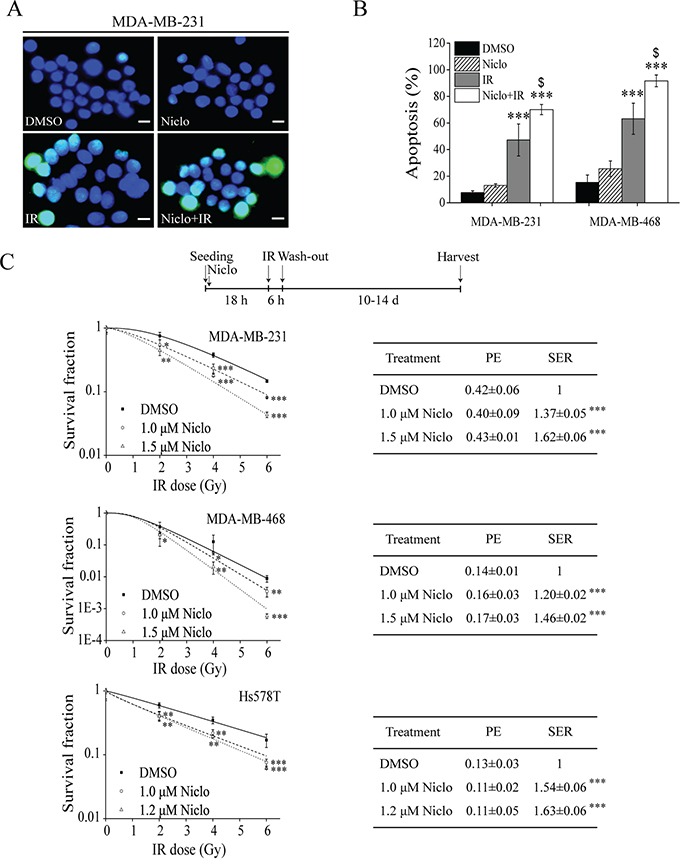
Niclosamide sensitizes TNBC cells to IR **A, B.** MDA-MB-231 cells were treated with γ-ray IR (6 Gy) and MDA-MB-468 cells were treated with X-ray IR (8 Gy) in the absence or presence of niclosamide (1.5 μM) for 24 h. Levels of apoptosis were examined by TUNEL staining at 48 h post-irradiation (400× magnification; Scale bar, 10 μm). Values are averages of three independent experiments with the standard deviations indicated by error bars. ^***^P<0.001 versus corresponding cells treated with DMSO control; ^$^p<0.05 versus corresponding cells treated with IR alone. **C.** MDA-MB-231, MDA-MB-468 and Hs578T cells were seeded into 60-mm dishes in duplicate, and were treated with γ-ray IR at indicated doses in the absence or presence of niclosamide at indicated concentrations for 24 h. After incubation for 10 to 14 days, the colonies with more than 50 cells were counted. The plating efficiency (PE) and the sensitizer enhancement ratio (SER) were determined as described in MATERIALS AND METHODS. Values are averages of three independent experiments with the standard deviations indicated by error bars. ^*^P<0.05, ^**^P<0.01, ^***^P<0.001 versus corresponding control cells. Niclo, niclosamide.

Given that niclosamide can inhibit IR-induced Wnt/β-catenin signaling in TNBC cells, we then examined whether niclosamide sensitizes TNBC cells to IR. Initially, we found that the cell viability IC_50_ values of niclosamide on MDA-MB-231 and Hs578T cells after 24 h treatment were 13.63±0.43 and 25.32±0.54 μM, respectively. Therefore, we tested niclosamide at 1.0, 1.2 and 1.5 μM for 24 h, which were not cytotoxic (less than 20% of the IC_50_ values) and had no effects on the plating efficiency (PE) of TNBC cells. It was found that niclosamide at 1.5 μM significantly enhanced IR-induced apoptosis in MDA-MB-231 and MDA-MB-468 cells (Figure 3A and 3B). Importantly, pretreatment of niclosamide at 1.0, 1.2 or 1.5 μM for 24 h significantly sensitized MDA-MB-231, MDA-MB-468 and Hs578T cells to IR with the sensitizer enhancement ratio (SER) between 1.20 to 1.63 (Figure 3C).

In addition, cell cycle analysis indicated that niclosamide blocked MDA-MB-231 cells at G1 phase and MDA-MB-468 cells at G2/M phase, and had little effect on cell cycle phases of Hs578T cells (Supplemental Figure S2), suggesting that the radiosensitizing effects of niclosamide in TNBC cells is unrelated to the blockage of mitotic cell cycle. Furthermore, we found that niclosamide was unable to sensitize non-TNBC T-47D and MCF-7 cells to IR (Supplemental Figure S3), suggesting that niclosamide specifically sensitize TNBC cells to IR.

### Niclosamide suppresses β-catenin-induced radioresistance in TNBC cells

**Figure 4:**
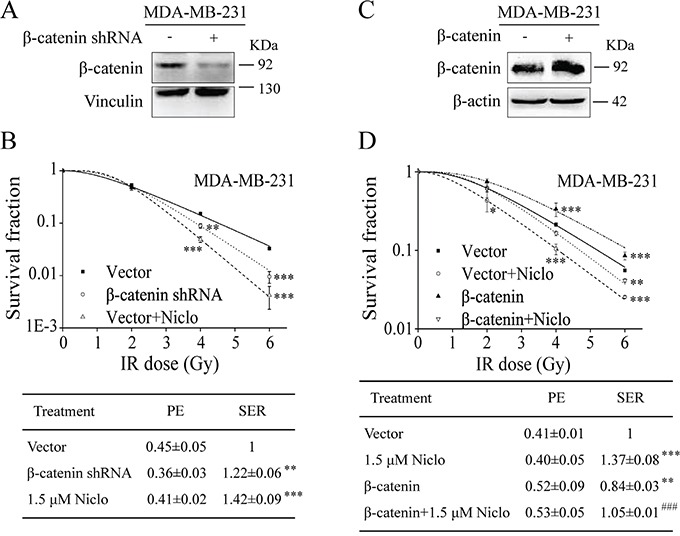
Niclosamide suppresses β-catenin-induced radioresistance in TNBC cells **A.** MDA-MB-231 cells were transfected with β-catenin shRNA or control vector. After 48 h incubation, the levels of β-catenin were examined by Western blotting. **B.** MDA-MB-231 cells in 60-mm dishes in duplicate were transfected with control vector, β-catenin shRNA, or control vector with niclosamide treatment, and treated with γ-ray IR at indicated doses. The colonies with more than 50 cells were counted after 12 to 14 days. **C.** MDA-MB-231 cells were transfected with β-catenin cDNA or vector. After 48 h incubation, the levels of β-catenin were examined by Western blotting. **D.** MDA-MB-231 cells in 60-mm dishes in duplicate were transfected with β-catenin cDNA or vector, and treated with γ-ray IR at indicated doses in the absence or presence of niclosamide (1.5 μM) for 24 h. After incubation for 10 to 14 days, the colonies with more than 50 cells were counted. PE and SER were determined as described in MATERIALS AND METHODS. Values are averages of three independent experiments with the standard deviations indicated by error bars. For SF curve, ^*^P<0.05, ^**^P<0.01, ^***^P<0.001 versus corresponding cells treated with DMSO control. For SER value, ^**^P<0.01, ^***^P<0.001 versus corresponding cells transfected with control vector and treated with DMSO; ^###^P<0.001 versus corresponding cells transfected with β-catenin plasmid. Niclo, niclosamide.

β-catenin is a central component in the Wnt/β-catenin signaling pathway. The binding of Wnt protein to its receptor Fzd and co-receptor LRP6 results in stabilization of cytosolic β-catenin, which then translocates into the nucleus where it interacts with T-cell factor/lymphoid enhancing factor (TCF/LEF) to induce the expression of downstream target genes [3–5]. To confirm that niclosamide-induced radiosensitization is associated with its ability to inhibit Wnt/β-catenin signaling, we tested whether β-catenin modulates the radiosensitivity of TNBC cells. We found that knockdown of β-catenin by shRNA significantly sensitized MDA-MB-231 cells to IR (Figure 4A and 4B), whereas overexpression of β-catenin reduced radiosensitivity of MDA-MB-231 cells (Figure 4C and 4D). Notably, niclosamide was able to overcome β-catenin overexpression-induced radioresistance of MDA-MB-231 cells (Figure 4D). Together, these results suggest that the inhibition of Wnt/β-catenin signaling by niclosamide contributes its radiosensitizing effects in TNBC cells.

### Niclosamide suppresses Wnt3a-induced Wnt/β-catenin signaling in TNBC cells and prevents Wnt3a-induced radioresistance

**Figure 5:**
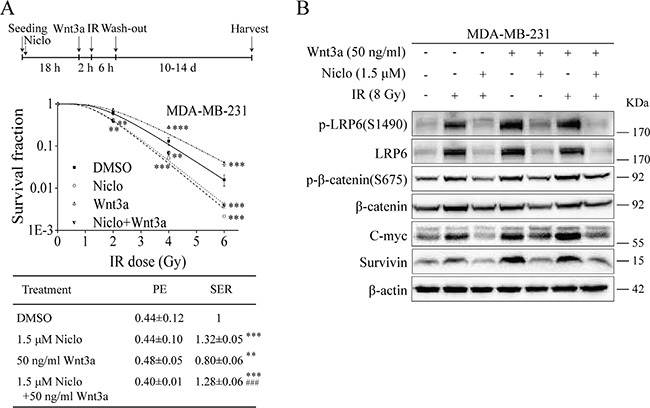
Niclosamide suppresses Wnt3a-induced Wnt/β-catenin signaling in TNBC cells and prevents Wnt3a-induced radioresistance **A.** MDA-MB-231 cells were seeded into 60-mm dishes in triplicate, and were treated with X-ray IR at indicated doses in the absence or presence of niclosamide (1.5 μM) for 24 h and Wnt3a (50 ng/ml) for 8 h. After incubation for 10 to 14 days, the colonies with more than 50 cells were counted. PE and SER were determined as described in MATERIALS AND METHODS. Values are averages of three independent experiments with the standard deviations indicated by error bars. ^**^P<0.01, ^***^P<0.001 versus corresponding cells treated with DMSO control. ^###^P<0.001 versus corresponding cells treated with Wnt3a alone. **B.** MDA-MB-231 cells were seeded into 60-mm dishes, and were treated with X-ray IR (8Gy) in the absence or presence of niclosamide (1.5 μM) for 24 h and Wnt3a (50 ng/ml) for 8 h. Levels of Wnt3a, p-LRP6 (S1490), LRP6, p-β-catenin (S675), β-catenin, C-myc and survivin were analyzed by Western blotting. All the samples were also probed with anti-β-actin antibody to verify equal loading. Niclo, niclosamide.

Wnt3a can induce Wnt/β-catenin signaling after its binding to Fzd and LRP6. Given that Wnt3a expression was up-regulated in TNBC cells after IR (Figure 1), we then examined whether niclosamide-induced radiosensitization is associated with its ability to inhibit Wnt3a-induced Wnt/β-catenin signaling in TNBC cells. It was found that treatment of recombinant Wnt3a protein at 50 ng/ml resulted in a decrease of radiosensitivity of MDA-MB-231 cells (Figure 5A), and that niclosamide was able to prevent Wnt3a-induced radioresistance of MDA-MB-231 cells (Figure 5A). As expected, both Wnt3a and IR (8 Gy) activated Wnt/β-catenin signaling, which was inhibited by niclosamide (1.5 μM) in MDA-MB-231 cells (Figure 5B).

### Effect of niclosamide and IR on established MDA-MB-231 tumors

**Figure 6:**
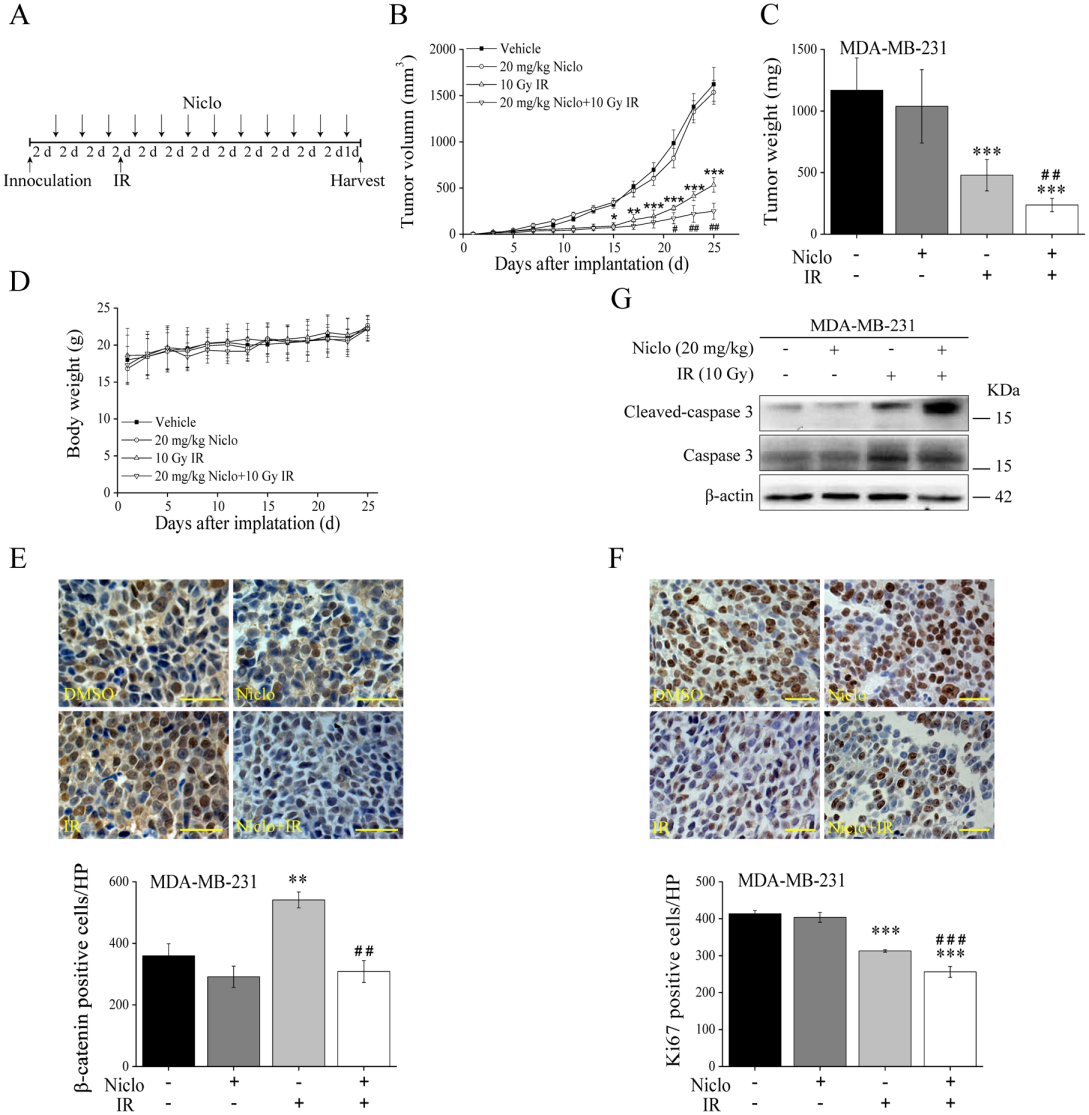
*In vivo* antitumor efficacy of niclosamide and IR in the TNBC MDA-MB-231 xenograft mouse model **A.** The treatment schedule of niclosamide and IR. **B.** Growth curve of tumor of nude mice treated with vehicle (DMSO:Tween 80:H_2_O= 3:4:8), niclosamide (20 mg/kg/dose), IR (10 Gy γ-ray at day 7), and niclosamide plus IR as described in MATERIALS AND METHODS. **C.** Nude mice were sacrificed and the tumors excised and weighed at the end of experiments. **D.** Body weights of nude mice treated with vehicle, niclosamide, IR, and niclosamide plus IR at the dosage and schedule as described in (B). **E.** Immunohistochemical staining with anti-β-catenin antibody of MDA-MB-231 tumors (400×magnification; Scale bar, 25 μm). **F.** Immunohistochemical staining with anti-Ki67 antibody of MDA-MB-231 tumors (400×magnification; Scale bar, 25 μm). **G.** Levels of cleaved caspase-3 in MDA-MB-231 tumors were examined by Western blotting. Data shown in (B-F) are averages from five nude mice. ^*^P<0.05, ^**^P<0.01, ^***^P<0.001 versus nude mice treated with vehicle control; ^#^p<0.05, ^##^p<0.01, ^###^p<0.001 versus nude mice treated with IR alone. Niclo, niclosamide.

Having established that niclosamide suppresses Wnt/β-catenin signaling in TNBC cells and sensitizes TNBC cells to IR, we then examined the combinational effects of niclosamide and IR in an MDA-MB-231 tumor xenograft model. Animals received 12 intraperitoneal injections of 20 mg/kg of niclosamide every other day, or a single 10 Gy γ-ray local irradiation of tumors at day 7 after tumor implantation, or both treatments and were compared with nude mice that received a vehicle control (Figure 6A). The nude mice that received γ-ray local irradiation alone had a significant inhibition of tumor growth (Figure 6B and 6C). While animals treated with niclosamide alone had no obvious effects on tumor growth, animals treated with the combination of niclosamide and γ-ray local irradiation had significant tumor growth inhibition compared with treated with γ-ray local irradiation (Figure 6B and 6C). On the other hand, the body weight of the treated nude mice, both with a single treatment and the combination did not decrease over time (Figure 6D). Immunohistochemical studies revealed that niclosamide was able to significantly inhibit β-catenin expression induced by IR in tumors (Figure 6E), suggesting that niclosamide suppresses IR-induced activation of Wnt/β-catenin signaling in tumors. We also characterized tumors for expression of cell proliferation marker Ki67, and found that γ-ray local irradiation of tumors resulted in a significant inhibition of Ki67 expression, which was further significantly decreased when the nude mice received the combinational treatment (Figure 6F). Moreover, Western blotting analysis of tumor tissues demonstrated that niclosamide enhanced IR-induced caspase-3 activation (Figure 6G), indicating the combinational effect of niclosamide and IR on TNBC cell apoptosis *in vivo*.

## DISCUSSION

Mounting evidences indicate that activation of Wnt/β-catenin signaling may be a key mechanism of radioresistance in cancer cells [17–19, 31–35]. It has been demonstrated that Wnt/β-catenin signaling plays an important role in the maintenance of radioresistant aldehyde dehydrogenase positive (ALDH^+^) prostate cancer cells [32], and that silencing of the Wnt transcription factor TCF4 sensitizes colorectal cancer cells to (chemo-) radiotherapy [33]. IR selectively enriches for breast cancer stem cells with highly activated Wnt/β-catenin signaling [17–19]. In the present study, we found that IR induced Wnt3a expression and activated Wnt/β-catenin signaling in TNBC cells. Moreover, treatment of Wnt3a protein or overexpression of β-catenin resulted in radioresistance of TNBC cells, whereas depletion of β-catenin sensitized TNBC cells to IR. Together, our findings indicate that the Wnt/β-catenin signaling pathway is an important pathway to control radiosensitivity in TNBC cells.

We noted that MDA-MB-468 cells were more radiosensitive than MDA-MB-231 and Hs578T cells (Figure 3C), which is consistent with the findings by other groups [36, 37]. It has been reported that the status of the retinoblastoma tumor suppressor (pRB) in TNBC cells dictates response to radiation treatment, and that pRB-negative TNBC cell lines such as MDA-MB-468 and BT549 are highly sensitive to IR compared to pRB-positive TNBC cell lines such as MDA-MB-231 and Hs578T [37]. The E2F1 transcription factor is a key downstream target of pRB, and a potent and specific inhibitor of Wnt/β-catenin signaling in colorectal cancer cells [38]. Wnt/β-catenin signaling activation is preferentially found in TNBC [7, 8, 12]. Therefore, further studies are necessary to determine whether E2F1 plays a role in Wnt/β-catenin signaling and radiosensitivity in TNBC cells.

Given that activation of the Wnt/β-catenin signaling pathway is a key radioprotective mechanism in irradiated cancer cells, therapies that specifically target this pathway could be a promising strategy to enhance the efficacy of radiotherapy. Indeed, inhibition of Wnt/β-catenin signaling by the tankyrase inhibitor XAV939 led to radiosensitization of prostate cancer cells and glioblastoma cells [31, 32]. In the present study, we found that niclosamide was able to sensitize TNBC cells to IR by inhibiting IR-induced Wnt/β-catenin signaling, indicating that the Wnt/β-catenin signaling pathway is not only important for acquiring radioresistance, but is also a therapeutic target for overcoming radioresistance in TNBC cells.

While survivin, a dual regulator of cancer cell proliferation and cell death, is a transcriptional target of Wnt/β-catenin signaling [39, 40], overexpression of survivin has been reported as the mediator of radioresistance, and inhibition of survivin is associated with increased radiosensitivity in several types of cancer [41–46]. In the present study, we demonstrated that IR induced Wnt3a expression, activated Wnt/β-catenin signaling and significantly enhanced survivin expression in TNBC cells. Importantly, niclosamide not only inhibited IR-inducedWnt3a expression and Wnt/β-catenin signaling activation in TNBC cells, but also suppressed the downstream target survivin expression and sensitized TNBC cells to IR. Together, our results suggest that survivin is a potential radioresistance factor in TNBC cells.

Niclosamide is an FDA approved anthelmintic drug, and has been used in humans for over 50 years [20, 21]. Recent studies found that niclosamide is a potential anticancer agent by inhibits multiple signaling pathways including the Wnt/β-catenin, mTORC1, STAT3, NF-κB and Notch signaling pathways in cancer cells [22]. In the present study, we demonstrated that niclosamide not only suppressed constitutive Wnt/β-catenin signaling, but also blocked IR-induced Wnt/β-catenin signaling in TNBC cells by inhibiting Wnt3a expression, LRP6 expression and LRP6 phosphorylation. Moreover, niclosamide could not only overcome β-catenin-induced radioresistance but also prevent Wnt3a-induced radioresistance in TNBC cells. Our findings indicate that the inhibition of Wnt/β-catenin signaling by niclosamide is associated with its radiosensitizing effects in TNBC cells. Recently, You *et al.* reported that niclosamide was able to reverse radioresistance of human lung cancer by blocking the STAT3/Bcl2/Bcl-XL survival signaling pathway [47]. Therefore, more studies are required to determine whether niclosamide inhibits other signaling pathways to contribute its radiosensitizing effects in TNBC cells in the future. Nevertheless, our findings provide rationale for further preclinical and clinical evaluation of niclosamide in TNBC management.

## MATERIALS AND METHODS

### Cell culture and transfection

TNBC MDA-MB-231, MDA-MB-468 and Hs578T cell lines were purchased from the Cell Bank of the Chinese Academy of Sciences (Shanghai, China). Cells were cultured in high glucose DMEM medium containing 10% fetal bovine serum (FBS), 2 mM of L-glutamine, 100 units/ml of penicillin, and 100 μg/ml of streptomycin under standard cell culture conditions at 37°C in a humidified atmosphere with 5% CO_2_. TNBC cells were transfected with β-catenin shRNA, β-catenin cDNA and the corresponding empty vector plasmids using Lipofectamine^®^ 2000 (Invitrogen/ThermoFisher Scientific, Grand Island, NY) according to manufacturer's specifications, respectively. β-catenin shRNA cloned in pLKO.1 vector, β-catenin cloned in pcDNA3 vector and the corresponding empty vector plasmids were obtained from Addgene (Cambridge, MA).

### Irradiation

TNBC cells were irradiated at room temperature using a ^137^Cs irradiator (Gammacell 40, MDS Nordion International Inc., Ottawa, Ontario, Canada) at a dose rate of 0.74 Gy/min or an X-Rad 320 biological irradiator (Precision X-Ray, Inc., North Branford, CT) at a dose rate of 1.83 Gy/min. Subcutaneous tumors at the right rear thighs of female athymic nude mice were local irradiated with rest of the body shielded with lead.

### Clonogenic assay

TNBC cells were plated into 60 mm culture dishes at various cell densities (2×10^2^-2×10^4^ cells/dish) and treated with niclosamide for 18 h. The cells were then exposed to IR at doses of 0, 2, 4 and 6 Gy γ-ray or X-ray. The cell culture media were changed at 6 h post-irradiation. The cells were further cultured for an additional 10~14 days, and colonies were fixed and stained with Giemsa. The colony is defined to consist of at least 50 cells. The plating efficiency (PE) was calculated as the number of colonies divided by the number of cells seeded, and the surviving fraction (SF) was determined by dividing the PE of the treated cells by the PE of the controls. The cell-survival curve was prepared with the multi-target single-hit model using Origin 9.2 software, and the mean lethal dose (D_0_) was calculated. The sensitizer enhancement ratio (SER) was determined as the D_0_ value of irradiated group divided by the D_0_ value of irradiation plus niclosamide-treated group [48].

### Western blotting

TNBC cells were plated into 60 mm culture dishes and treated with niclosamide for 18 h. The cells were then exposed to 6 Gy γ-ray or 8 Gy X-ray IR. After 6 h incubation, the cells were lysed in radioimmunoprecipitation assay (RIPA) buffer containing 1% protease inhibitor cocktail, 1% phosphatase inhibitor cocktail, 1 mM phenylmethylsulfonyl fluoride (PMSF) and 1 mM sodium orthovanadate at 4°C for 30 min. Equal quantities of protein were subjected to SDS–PAGE under reducing conditions, and transferred to the polyvinylidene fluoride (PVDF) membrane. Membranes were blocked with 5% non-fat milk in Tris-buffered saline and Tween 20 (TBST) for 1 h at room temperature, and incubated overnight at 4°C with anti-Wnt3a (1:1000, Abcam (Hong Kong) Ltd., HK, China), anti-p-LRP6 (S1490) (1:1000, Cell Signaling Technology, Danvers, MA), anti-LRP6 (1:1000, Cell Signaling Technology), anti-p-β-catenin (S675) (1:1000, Cell Signaling Technology), anti-β-catenin (1:1000, Cell Signaling Technology), anti-C-myc (1:5000, Epitomics, Burlingame, CA), anti-Survivin (1:5000, Epitomics), anti-β-actin (1:2000, HuaAn Biotechnology, Hangzhou, Zhejiang Province, China), and anti-Vinculin (1:10000, Abcam) antibodies diluted in the blocking buffer. After several washes with PBS containing 0.1% Tween 20, membranes were incubated in the blocking buffer with horseradish peroxide conjugated secondary antibody for 60 min at room temperature. After washing, the immunoreactive proteins were then detected using the ECL system. Blots were quantitated by densitometric analysis using a ChemiDoc XRS system (Bio-Rad Laboratories Inc., Hercules, CA) and normalized to a housekeeper gene β-actin or vinculin.

### Real-time RT-PCR assay

TNBC MDA-MB-231, MDA-MB-468 and Hs578T cells were plated into 60 mm culture dishes. Cells were then irradiated at room temperature using a ^137^Cs irradiator (Gammacell 40, MDS Nordion International Inc.) at a dose rate of 0.74 Gy/min. Total RNA was isolated from cell cultures using RNA-prep Pure Cell/Bacteria Kit (TIANGEN Biotech, Beijing, China). First-strand cDNA synthesis was performed in a 20 μl of reaction mixture containing 2 μg of total RNA with FastQuant RT Kit (TIANGEN). For analysis of Wnt3a, LRP6, β-catenin, C-myc and survivin expression, real-time RT-PCR was carried out using 2 μl of cDNA in a total volume of 20 μl after 40 cycles as described by TIANGEN SuperReal PreMix Plus kit, and β-actin was used as a control. The forward and reverse primers for β-actin are 5-TGACGTGGACATCCGCAAAG-3 and 5-CTGGAAGGTGGACAGCGAGG-3. The forward and reverse primers for Wnt3a are 5-ATCCTCTGC CTCAAATTCT-3 and 5-TTCGTCTAACTCCGTTGG-3. The forward and reverse primers for LRP6 are 5-GATTA TCCAGAAGGCATGGCAG-3 and 5-TCCCATCACCAT CTTCCA-3. The forward and reverse primers for β-catenin are 5-GTACGTCCATGGGTGGGACA-3 and 5-GGCTC CGGTACAACCTTCAACTA-3. The forward and reverse primers for C-myc are 5-CAGCTGCTTAGACGCTG GATTT-3 and 5-ACCGAGTCGTAGTCGAGGTCAT-3. The forward and reverse primers for survivin are 5-CCAC CGCATCTCTACATTCA-3 and 5-TATGTTCCTCTA TGGGGTCG-3. The polymerase chain reaction data were analyzed according to the 2^−ΔΔCT^ method.

### Immunofluorescent staining of β-catenin in TNBC cells

TNBC cells growing on coverslips were treated with niclosamide (1.5 μM) for 18 h. The cells were then exposed to 6 Gy γ-ray IR. After 6 h incubation, the cells were fixed in 4% paraformaldehyde, permeabilized with 0.5% Triton X-100, blocked with 10% (v:v) FBS in phosphate-buffered saline (PBS), labeled with rabbit monoclonal β-catenin (6B3) antibody (1:200, Cell Signaling Technology), and detected with Alexa Fluor 488 donkey anti-rabbit IgG (1:500, Molecular Probes/ThermoFisher Scientific, Grand Island, NY). The nuclei were counter stained with DAPI (Santa Cruz Biotechnology Inc., Santa Cruz, CA), and the images were examined under an Olympus BX51 fluorescent microscope. The mean fluorescence intensity of nuclear β-catenin staining was calculated out of a total number of 200 cells per sample, and was normalized to that of a control sample for the calculation of the relative fluorescence intensity.

### Apoptosis detection

TNBC cells growing on coverslips were treated with niclosamide (1.5 μM) for 18 h. The cells were then exposed to 6 Gy γ-ray or 8 Gy X-ray IR. After 48 h incubation, the cells were subjected to terminal deoxynucleotidyl transferase-mediated dUTP biotin nick end labeling (TUNEL) staining using a TUNEL Apoptosis Detection Kit (Vazyme Biotech, Nanjing, Jiangsu Province, China) for *in situ* detection of apoptosis according to the manufacturer's instructions. The cells were counter-stained with mounting medium containing DAPI (Santa Cruz Biotechnology), and the FITC-labeled TUNEL-positive cells were imaged using an Olympus BX51 fluorescent microscope. The percentage of TUNEL-positive cells was calculated out of a total number of 100 cells per sample that were detected in different and randomly chosen microscopic fields.

### Antitumor efficacy studies

Female athymic nude mice at 4-5 weeks of age were purchase from Shanghai Silaike Laboratory Animal Co. Ltd. (Shanghai, China) and housed under specific-pathogen-free condition. The nude mice received autoclaved diet and water ad libitum. MDA-MB-231 cells (5×10^6^ cells) were suspended in 0.1 ml of PBS, and injected subcutaneously into the right rear thighs of the nude mice. The day of tumor implantation was designated as day 0, and the nude mice were randomly assigned into four groups (5 nude mice per group). Niclosamide at a dosage of 20 mg/kg/dose was given every other day by intraperitoneal injection. A single 10 Gy γ-ray local irradiation of tumor was given at day 7. Tumor dimensions and body weights were measured every other day. Tumor volume was determined by caliper measurements (mm) and using the formula for an ellipsoid sphere: L × W^2^/2 (mm^3^), where L and W refer to the larger and smaller perpendicular measurements, respectively. Nude mice were sacrificed by decapitation and tumors were immediately removed after death. All animal protocols were reviewed and approved by the Animal Research Ethics Committee of School of Pharmacy of Fudan University prior to experimentation.

### Immunohistochemistry staining for tumor tissues

The sections from paraffin-embedded tumor tissues were analyzed by immunohistochemical (IHC) staining using anti-β-catenin (6B3) antibody (1:200, Cell Signaling Technology) or anti-Ki-67 (8D5) antibody (1:400, Cell Signaling Technology), respectively. At least 5 random fields of each section were examined using an Olympus IX51 microscope. The average number of positive cells per field was determined from five independent tumor samples.

### Statistical analysis

All data are expressed as the mean ± standard deviation. Statistical analyses of the data were performed using SPSS 18.0 software (SPSS Inc., Chicago, IL). Student's *t*-test was applied to analyze the difference between two means. *P* value of less than 0.05 was considered to indicate statistical significance.

## SUPPLEMENTARY MATERIALS AND METHODS



## References

[1] Carey L, Winer E, Viale G, Cameron D, Gianni L (2010). Triple-negative breast cancer: disease entity or title of convenience?. Nat Rev Clin Oncol.

[2] Pal SK, Childs BH, Pegram M (2011). Triple negative breast cancer: unmet medical needs. Breast Cancer Res Treat.

[3] Anastas JN, Moon RT (2013). WNT signalling pathways as therapeutic targets in cancer. Nat Rev Cancer.

[4] Clevers H, Nusse R (2012). Wnt/beta-catenin signaling and disease. Cell.

[5] Kahn M (2014). Can we safely target the WNT pathway?. Nat Rev Drug Discov.

[6] Lin SY, Xia W, Wang JC, Kwong KY, Spohn B, Wen Y, Pestell RG, Hung MC (2000). Beta-catenin, a novel prognostic marker for breast cancer: its roles in cyclin D1 expression and cancer progression. Proc Natl Acad Sci U S A.

[7] Khramtsov AI, Khramtsova GF, Tretiakova M, Huo D, Olopade OI, Goss KH (2010). Wnt/beta-catenin pathway activation is enriched in basal-like breast cancers and predicts poor outcome. Am J Pathol.

[8] Geyer FC, Lacroix-Triki M, Savage K, Arnedos M, Lambros MB, MacKay A, Natrajan R, Reis-Filho JS (2011). beta-Catenin pathway activation in breast cancer is associated with triple-negative phenotype but not with CTNNB1 mutation. Mod Pathol.

[9] Yang L, Wu X, Wang Y, Zhang K, Wu J, Yuan YC, Deng X, Chen L, Kim CC, Lau S, Somlo G, Yen Y (2011). FZD7 has a critical role in cell proliferation in triple negative breast cancer. Oncogene.

[10] Lindvall C, Zylstra CR, Evans N, West RA, Dykema K, Furge KA, Williams BO (2009). The Wnt co-receptor Lrp6 is required for normal mouse mammary gland development. PLoS One.

[11] Liu CC, Prior J, Piwnica-Worms D, Bu G (2010). LRP6 overexpression defines a class of breast cancer subtype and is a target for therapy. Proc Natl Acad Sci U S A.

[12] King TD, Suto MJ, Li Y (2012). The Wnt/beta-catenin signaling pathway: a potential therapeutic target in the treatment of triple negative breast cancer. J Cell Biochem.

[13] Abdulkarim BS, Cuartero J, Hanson J, Deschenes J, Lesniak D, Sabri S (2011). Increased risk of locoregional recurrence for women with T1-2N0 triple-negative breast cancer treated with modified radical mastectomy without adjuvant radiation therapy compared with breast-conserving therapy. J Clin Oncol.

[14] Wang J, Shi M, Ling R, Xia Y, Luo S, Fu X, Xiao F, Li J, Long X, Wang J, Hou Z, Chen Y, Zhou B, Xu M (2011). Adjuvant chemotherapy and radiotherapy in triple-negative breast carcinoma: a prospective randomized controlled multi-center trial. Radiother Oncol.

[15] Steward LT, Gao F, Taylor MA, Margenthaler JA (2014). Impact of radiation therapy on survival in patients with triple-negative breast cancer. Oncol Lett.

[16] Valerie K, Yacoub A, Hagan MP, Curiel DT, Fisher PB, Grant S, Dent P (2007). Radiation-induced cell signaling: inside-out and outside-in. Mol Cancer Ther.

[17] Woodward WA, Chen MS, Behbod F, Alfaro MP, Buchholz TA, Rosen JM (2007). WNT/beta-catenin mediates radiation resistance of mouse mammary progenitor cells. Proc Natl Acad Sci U S A.

[18] Chen MS, Woodward WA, Behbod F, Peddibhotla S, Alfaro MP, Buchholz TA, Rosen JM (2007). Wnt/beta-catenin mediates radiation resistance of Sca1+ progenitors in an immortalized mammary gland cell line. J Cell Sci.

[19] Zhang M, Atkinson RL, Rosen JM (2010). Selective targeting of radiation-resistant tumor-initiating cells. Proc Natl Acad Sci U S A.

[20] Andrews P, Thyssen J, Lorke D (1982). The biology and toxicology of molluscicides, Bayluscide. Pharmacol Ther.

[21] Al-Hadiya BM (2005). Niclosamide: comprehensive profile. Profiles Drug Subst Excip Relat Methodol.

[22] Li Y, Li PK, Roberts MJ, Arend RC, Samant RS, Buchsbaum DJ (2014). Multi-targeted therapy of cancer by niclosamide: A new application for an old drug. Cancer Lett.

[23] Chen M, Wang J, Lu J, Bond MC, Ren XR, Lyerly HK, Barak LS, Chen W (2009). The anti-helminthic niclosamide inhibits Wnt/Frizzled1 signaling. Biochemistry.

[24] Sack U, Walther W, Scudiero D, Selby M, Kobelt D, Lemm M, Fichtner I, Schlag PM, Shoemaker RH, Stein U (2011). Novel effect of antihelminthic Niclosamide on S100A4-mediated metastatic progression in colon cancer. J Natl Cancer Inst.

[25] Lu W, Lin C, Roberts MJ, Waud WR, Piazza GA, Li Y (2011). Niclosamide suppresses cancer cell growth by inducing Wnt co-receptor LRP6 degradation and inhibiting the Wnt/beta-catenin pathway. PLoS One.

[26] Osada T, Chen M, Yang XY, Spasojevic I, Vandeusen JB, Hsu D, Clary BM, Clay TM, Chen W, Morse MA, Lyerly HK (2011). Antihelminth compound niclosamide downregulates Wnt signaling and elicits antitumor responses in tumors with activating APC mutations. Cancer Res.

[27] Londono-Joshi AI, Arend RC, Aristizabal L, Lu W, Samant RS, Metge BJ, Hidalgo B, Grizzle WE, Conner M, Forero-Torres A, Lobuglio AF, Li Y, Buchsbaum DJ (2014). Effect of niclosamide on basal-like breast cancers. Mol Cancer Ther.

[28] Hino S, Tanji C, Nakayama KI, Kikuchi A (2005). Phosphorylation of beta-catenin by cyclic AMP-dependent protein kinase stabilizes beta-catenin through inhibition of its ubiquitination. Mol Cell Biol.

[29] Taurin S, Sandbo N, Qin Y, Browning D, Dulin NO (2006). Phosphorylation of beta-catenin by cyclic AMP-dependent protein kinase. J Biol Chem.

[30] Chandra A, Lin T, Zhu J, Tong W, Huo Y, Jia H, Zhang Y, Liu XS, Cengel K, Xia B, Qin L (2015). PTH1-34 blocks radiation-induced osteoblast apoptosis by enhancing DNA repair through canonical Wnt pathway. J Biol Chem.

[31] Kim Y, Kim KH, Lee J, Lee YA, Kim M, Lee SJ, Park K, Yang H, Jin J, Joo KM, Lee J, Nam DH (2012). Wnt activation is implicated in glioblastoma radioresistance. Lab Invest.

[32] Cojoc M, Peitzsch C, Kurth I, Trautmann F, Kunz-Schughart LA, Telegeev GD, Stakhovsky EA, Walker JR, Simin K, Lyle S, Fuessel S, Erdmann K, Wirth MP, Krause M, Baumann M, Dubrovska A (2015). Aldehyde Dehydrogenase Is Regulated by beta-Catenin/TCF and Promotes Radioresistance in Prostate Cancer Progenitor Cells. Cancer Res.

[33] Kendziorra E, Ahlborn K, Spitzner M, Rave-Frank M, Emons G, Gaedcke J, Kramer F, Wolff HA, Becker H, Beissbarth T, Ebner R, Ghadimi BM, Pukrop T, Ried T, Grade M (2011). Silencing of the Wnt transcription factor TCF4 sensitizes colorectal cancer cells to (chemo-) radiotherapy. Carcinogenesis.

[34] Chang HW, Roh JL, Jeong EJ, Lee SW, Kim SW, Choi SH, Park SK, Kim SY (2008). Wnt signaling controls radiosensitivity via cyclooxygenase-2-mediated Ku expression in head and neck cancer. Int J Cancer.

[35] Kim HS, Kim SC, Kim SJ, Park CH, Jeung HC, Kim YB, Ahn JB, Chung HC, Rha SY (2012). Identification of a radiosensitivity signature using integrative metaanalysis of published microarray data for NCI-60 cancer cells. BMC Genomics.

[36] Lee KM, Choi EJ, Kim IA (2011). microRNA-7 increases radiosensitivity of human cancer cells with activated EGFR-associated signaling. Radiother Oncol.

[37] Robinson TJ, Liu JC, Vizeacoumar F, Sun T, Maclean N, Egan SE, Schimmer AD, Datti A, Zacksenhaus E (2013). RB1 status in triple negative breast cancer cells dictates response to radiation treatment and selective therapeutic drugs. PLoS One.

[38] Morris EJ, Ji JY, Yang F, Di Stefano L, Herr A, Moon NS, Kwon EJ, Haigis KM, Naar AM, Dyson NJ (2008). E2F1 represses beta-catenin transcription and is antagonized by both pRB and CDK8. Nature.

[39] Kim PJ, Plescia J, Clevers H, Fearon ER, Altieri DC (2003). Survivin and molecular pathogenesis of colorectal cancer. Lancet.

[40] Ma H, Nguyen C, Lee KS, Kahn M (2005). Differential roles for the coactivators CBP and p300 on TCF/beta-catenin-mediated survivin gene expression. Oncogene.

[41] Rodel F, Frey B, Leitmann W, Capalbo G, Weiss C, Rodel C (2008). Survivin antisense oligonucleotides effectively radiosensitize colorectal cancer cells in both tissue culture and murine xenograft models. Int J Radiat Oncol Biol Phys.

[42] Asanuma K, Moriai R, Yajima T, Yagihashi A, Yamada M, Kobayashi D, Watanabe N (2000). Survivin as a radioresistance factor in pancreatic cancer. Jpn J Cancer Res.

[43] Rodel F, Hoffmann J, Distel L, Herrmann M, Noisternig T, Papadopoulos T, Sauer R, Rodel C (2005). Survivin as a radioresistance factor, and prognostic and therapeutic target for radiotherapy in rectal cancer. Cancer Res.

[44] Rodel C, Haas J, Groth A, Grabenbauer GG, Sauer R, Rodel F (2003). Spontaneous and radiation-induced apoptosis in colorectal carcinoma cells with different intrinsic radiosensitivities: survivin as a radioresistance factor. Int J Radiat Oncol Biol Phys.

[45] Chakravarti A, Zhai GG, Zhang M, Malhotra R, Latham DE, Delaney MA, Robe P, Nestler U, Song Q, Loeffler J (2004). Survivin enhances radiation resistance in primary human glioblastoma cells via caspase-independent mechanisms. Oncogene,.

[46] Ogura A, Watanabe Y, Iizuka D, Yasui H, Amitani M, Kobayashi S, Kuwabara M, Inanami O (2008). Radiation-induced apoptosis of tumor cells is facilitated by inhibition of the interaction between Survivin and Smac/DIABLO. Cancer Lett.

[47] You S, Li R, Park D, Xie M, Sica GL, Cao Y, Xiao ZQ, Deng X (2014). Disruption of STAT3 by niclosamide reverses radioresistance of human lung cancer. Mol Cancer Ther.

[48] Barazzuol L, Jeynes JC, Merchant MJ, Wera AC, Barry MA, Kirkby KJ, Suzuki M (2015). Radiosensitization of glioblastoma cells using a histone deacetylase inhibitor (SAHA) comparing carbon ions with X-rays. Int J Radiat Biol.

