# Supplementary material for: Niclosamide sensitizes triple-negative breast cancer cells to ionizing radiation in association with the inhibition of Wnt/β-catenin signaling
Source: Oncotarget. 2016 May 30;7(27):42126–38. doi: 10.18632/oncotarget.9704 (PMC5173121; doi:10.18632/oncotarget.9704)
Supplement: Supplementary file 1 [file oncotarget-07-42126-s001.pdf]

Niclosamide sensitizes triple-negative breast cancer cells to ionizing radiation in association with the inhibition of Wnt/β-catenin signaling

SUPPLEMENTARY DATA

SUPPLEMENTAL MATERIALS AND METHODS

Cell cycle analysis

TNBC MDA-MB-231, MDA-MB-468 and Hs578T cells were treated with niclosamide (1.5 μM) for 24 h. Cells were then harvested and fixed with cold 70% ethanol for overnight, and analyzed with the cell cycle analysis kit (Beyotime Institute of Biotechnology, Haimen, Jiangsu Province, China). DNA content was measured by a Gallios flow cytometer (Beckman Coulter Inc., Brea, CA, USA). Data were analyzed to get the percentages of cells in G1, S and G2/M phases.

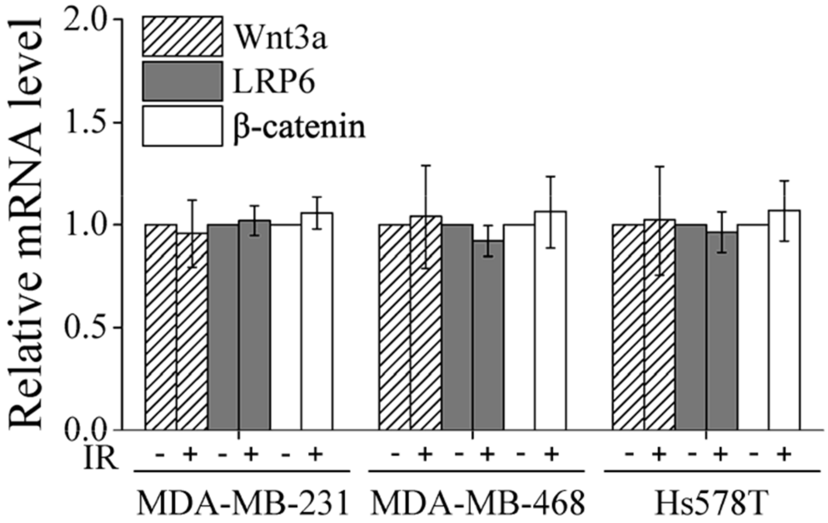

Supplementary Figure S1: Effect of IR on Wnt3a, LRP6 and β-catenin mRNA levels in TNBC cells. MDA-MB-231, MDA-MB-468 and Hs578T cells were irradiated with 6 Gy γ-rays. After 6 h incubation, the transcriptional levels of Wnt3a, LRP6, β-catenin were measured by real-time RT-PCR and normalized to the levels of β-actin. Data are averages of three independent experiments with the standard deviations indicated by error bars.

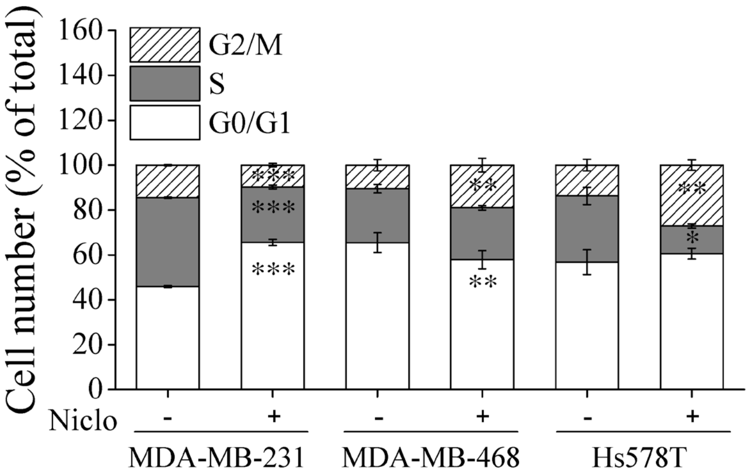

Supplementary Figure S2: Effect of niclosamide on cell cycle of TNBC cells. MDA-MB-231, MDA-MB-468 and Hs578T cells were treated with 1.5 µM niclosamide for 24 h, and cell cycle was analyzed by flow cytometry. Values are averages of three independent experiments with the standard deviations indicated by error bars. $^{**}P<0.01$, $^{***}P<0.001$ versus corresponding cells treated with DMSO.

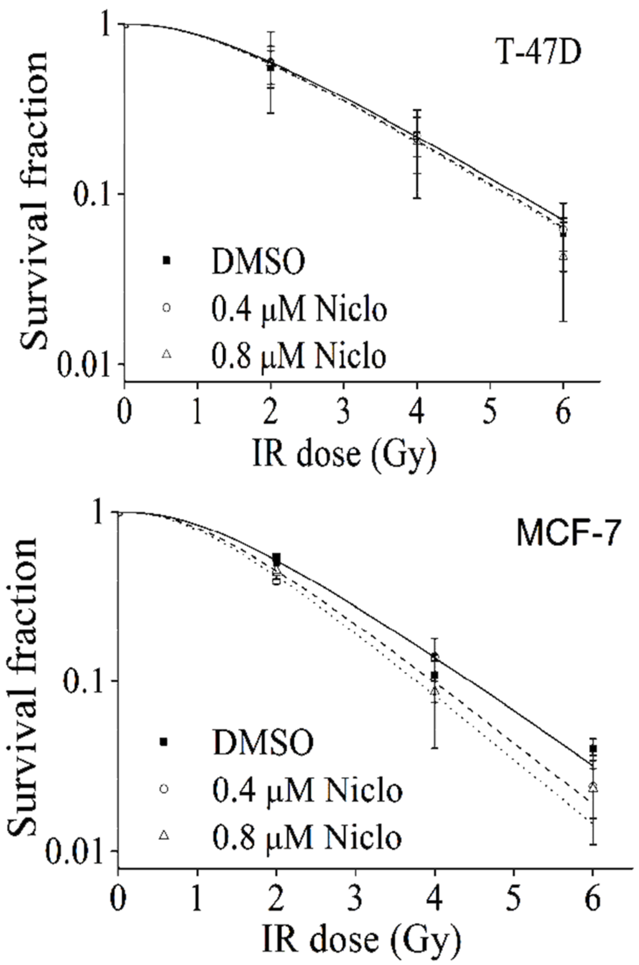

Treatment	PE	SER
DMSO	0.18±0.02	1
0.4 µM Niclo	0.21±0.10	0.96±0.04
0.8 µM Niclo	0.22±0.02	0.99±0.03

Treatment	PE	SER
DMSO	0.18±0.02	1
0.4 µM Niclo	0.19±0.01	1.12±0.06
0.8 µM Niclo	0.19±0.02	1.18±0.10

Supplementary Figure S3: Niclosamide was unable to sensitize non-TNBC T-47D and MCF-7 cells to IR. T-47D and MCF-7 cells were seeded into 60-mm dishes in triplicate, and were irradiated with γ-ray at indicated doses in the absence or presence of niclosamide (0.4 and 0.8 µM, ≤ 20% of cell viability IC_{50} values after 24 h treatment) for 24 h. After incubation for 10 to 14 days, the colonies with more than 50 cells were counted. The plating efficiency (PE) and the sensitizer enhancement ratio (SER) were determined as described in MATERIALS AND METHODS. Values are averages of three independent experiments with the standard deviations indicated by error bars.
